# Association between long-term exposure to ambient particulate matter and pulmonary function among men and women in typical areas of South and North China

**DOI:** 10.3389/fpubh.2023.1170584

**Published:** 2023-05-12

**Authors:** Qihang Liu, Li Pan, Ting Yang, Qiong Ou, Zhiwei Sun, Huijing He, Yaoda Hu, Ji Tu, Binbin Lin, Miaochan Lao, Chang Liu, Baicun Li, Yajiao Fan, Hongtao Niu, Longlong Wang, Guangliang Shan

**Affiliations:** ^1^Department of Epidemiology and Statistics, Institute of Basic Medical Sciences, School of Basic Medicine, Chinese Academy of Medical Sciences, Peking Union Medical College, Beijing, China; ^2^China-Japan Friendship Hospital, National Center for Respiratory Medicine, Institute of Respiratory Medicine, Chinese Academy of Medical Sciences, National Clinical Research Center for Respiratory Diseases, Beijing, China; ^3^Sleep Center, Department of Pulmonary and Critical Care Medicine, Guangdong Provincial People’s Hospital, Guangdong Academy of Medical Sciences, Guangdong Provincial Geriatrics Institute, Guangzhou, China; ^4^Department of Preventive Medicine, School of Public Health, Hebei University, Baoding, Hebei, China

**Keywords:** PM_1_, PM_2.5_, PM_10_, pulmonary function, restrictive ventilatory dysfunction

## Abstract

**Background:**

Studies comparing the effects of different sizes and concentrations of ambient particulate matter (PM) on pulmonary function in different regions and sexes remain sparse.

**Objectives:**

To investigate the associations of different sizes and levels of long-term ambient PM exposure with pulmonary function among people of different sexes in typical areas of South and North China.

**Methods:**

In 2021, a total of 1,592 participants aged 20–73 years were recruited to participate in the pulmonary function test from the baseline survey of the Diverse Life-Course Cohort (DLCC) in typical areas of Guangdong Province and Hebei Province. The three-year (2018–2020) average ambient PM concentrations were assessed from the ChinaHighPM_1_ dataset, ChinaHighPM_2.5_ dataset and ChinaHighPM_10_ dataset. Mean differences in pulmonary function were used in multilevel models for different regions and sexes.

**Results:**

We discovered significant associations of ambient PM exposure with reduced forced vital capacity (FVC) and increased forced expiratory volume in 1 s/forced vital capacity ratio (FEV_1_/FVC) among men and lower levels of FEV_1_ and FVC among women, such that a 5-μg/m^3^ concentration increase in PM_1_, PM_2.5_, and PM_10_ was associated with decreases in FVC of 122.1 ml (95% confidence interval (CI): 30.8, 213.4), 54.6 ml (95% CI: 15.8, 93.3) and 42.9 ml (95% CI: 12.7, 73.1) and increases in FEV_1_/FVC of 2.2% (95% CI: 0.6, 3.9), 1.1% (95% CI: 0.4, 1.9) and 0.9% (95% CI: 0.3, 1.5) among men and decreases in FEV_1_ of 51.1 ml (95% CI: 9.7, 92.4), 21.6 ml (95% CI: 4.3, 38.9) and 16.7 ml (95% CI: 3.3, 30.1) and in FVC of 77.8 ml (95% CI: 10.0, 145.6), 38.7 ml (95% CI: 9.0, 68.5) and 31.1 ml (95% CI: 8.1, 54.1) among women in Hebei Province. There was no association between ambient PM and pulmonary function in Guangdong Province.

**Conclusion:**

Long-term exposure to different sizes and concentrations of ambient PM were associated with FEV_1_ and FVC among men and women differently. The impact of ambient PM on FVC should be of greater concerned.

## 1. Introduction

Particulate matter (PM) is a major risk factor for disease burden worldwide and long-term exposure to ambient PM is greatly associated with cardiovascular and respiratory diseases (1–4). In addition to resulting in multisystem disorders, including respiratory diseases, ambient PM seems to reduce pulmonary function directly (5–8). In general, PM_10_ is mostly released into the atmosphere through mechanical processes like the production of construction dust, wind-blown mineral soil and sea-salt, PM_2.5_ is largely generated by human activities, such as power generation, industrial manufacturing, and residue emissions. While some sources are common, PM_1_ mainly originates from direct emissions during the combustion process (9–11). The effect of PM on pulmonary function is associated with size because finer PM is more likely to reach the terminal bronchi (12, 13). In addition, smaller particles cause greater cytotoxic effects and inflammation (14). Thus, finer PM may have a greater impact on pulmonary function.

Pulmonary function is an objective indicator that reflects respiratory health, which can be measured by pulmonary function instruments. Portable and easy to operate, pulmonary function instruments are often used for large-scale population surveys to evaluate pulmonary health and screen for respiratory diseases. According to the 2019 Global Initiative for Chronic Obstructive Lung Disease guidelines, a postbronchodilator forced expiratory volume in 1 s/forced vital capacity ratio (FEV_1_/FVC) < 0.70 is defined as chronic obstructive pulmonary disease (COPD) (15). Asthma, pulmonary fibrosis, lung cancer and other respiratory diseases are also accompanied by decreased pulmonary function. A study also indicated that decreased pulmonary function is a mediator of cardiopulmonary death (16).

Thanks to publicly available *in situ* measurements of PM_2.5_ (particulate matter ≤2.5 μm in aerodynamic diameter) and PM_10_ (particulate matter ≤10 μm in aerodynamic diameter), there have been several studies on the associations between long-term exposure to PM_2.5_ and PM_10_ and pulmonary function. All forced expiratory volume parameters decrease with increment in PM_2.5_ and PM_10_ in the UK and Taiwan; Increase in PM_2.5_ was associated with lower FEV_1_ and FVC, but association with FEV_1_/FVC ratio was weak or absent in the Northeastern United States; And a slight positive correlation between PM_2.5_ and FEV_1_ and FVC in some places of northwestern China (17–20). Although the findings were inconsistent, they all showed that pulmonary function declined at different levels with increasing PM_2.5_ and PM_10_ concentrations. Governments worldwide have taken measures to improve air quality. Since the implementation of the Air Pollution Prevention and Control Action Plan in the Beijing-Tianjin-Hebei region (BTH region, one of the most polluted areas in China) in 2013, air quality has greatly improved but was still not adequate until 2018 (21). Evidence of the association between ambient PM and pulmonary diseases in the BTH region is limited. Moreover, studies on PM_1_ are much rarer due to fewer ground monitoring stations. Presently, there are not large-scale population surveys on the relationship between PM_1_ (particulate matter ≤1 μm in aerodynamic diameter) and pulmonary function. Evidence suggests that PM_1_ exposure is associated with poorer pulmonary function in children and adolescents (22–24).In addition, regional and sex discrepancies in pulmonary function are substantial (25); however, few studies distinguished areas and sexes when exploring the association between ambient PM and pulmonary function and none of them compared the different effects of PM_1_, PM_2.5_ and PM_10_ on pulmonary function in the general population.

The BTH region is one of the world-class urban agglomerations in China that serves as the country’s political center and third largest economy. With 8.1% of China’s population in this region, it has witnessed rapid socioeconomic growth and urbanization, and, consequently caused severe air pollution and substantial changes in health-related behaviors (26). In Baoding (southern BTH region in North China), due to coal consumption and increasing numbers of private cars, ambient PM pollution is relatively serious. The annual average concentration of PM_2.5_ exceeded 50 μg/m^3^ for many years in Baoding (21), Hebei Province, which exceeded the World Health Organization (WHO) (27) Air Quality Guidelines (5 μg/m^3^) by >10-fold (2021). Nonetheless, with the World Geopark Baishishan Mountain, the air quality of Laiyuan County is far better than that of Baoding city. We selected mountainous regions, coastal cities and islands in Shantou and Meizhou, Guangdong Province (South China) as clean controls outside Baoding and the average annual concentration of PM_2.5_ is approximately 20 μg/m^3^ in Shantou and Meizhou every year. This provides a natural condition with two clean controls inside and outside Baoding city to explore the different associations between high-level and low-level ambient PM and pulmonary function. In this comparative study, research was conducted by using the baseline survey of the Chaoshan-Hakka-Baoding-general population cohort (CHB cohort) in the Diverse Life-Course Cohort (DLCC) in Shantou and Meizhou, Guangdong Province (South China) and Baoding, Hebei Province (North China) to investigate the different associations between high-level and low-level exposure to ambient PM and pulmonary function.

## 2. Methods

### 2.1. Study population

This comparative study was based on a baseline survey of the CHB cohort in the DLCC. Detailed information on the whole research has been described previously (28). Briefly, At Visit 2 of the DLCC, we singled out typical areas in Guangdong Province (South China) and Hebei Province (North China), including coastal Shantou city-Chenghai and Jinping, island-Nan’ao County, southern mountainous regions -Meizhou city and Jiaoling county, northern plains area-Baoding city and northern mountainous region-Laiyuan county. The newly enrolled areas have some unique characteristics in culture, dietary patterns, environmental risk factors (such as concentration of ambient PM), and noncommunicable chronic diseases (NCDs) prevalence, which enabled us to conduct comparative studies on the effects of different ambient pollution patterns on multiple health outcomes. The main aim of Visit 2 of the DLCC was to explore the associations of ambient pollutants and other environmental risk factors with NCDs in the general population in China.

Candidates who were 20-to 80-year-old permanent residents without severe physical or mental disease were included in the study sample. A total of 9,866 participants responded and completed the baseline survey. To ensure an adequate sample size and control for confounding factors related to pulmonary function, we selected as many nonsmoking participants under 50 years of age without a history of respiratory disease or an occupational history of high-risk respiratory disease as possible. If there were not enough participants in pulmonary function tests, we relaxed the criteria. Finally, only 1,592 participants aged 20–73 years whose pulmonary function parameters were valid were included.

Demographic characteristics, socioeconomic characteristics, health behavior, physician-diagnosed diseases and medication histories were collected via face-to-face questionnaire interviews by well-trained investigators. Height, weight, and pulmonary function were collected parameters from anthropometric measurements.

### 2.2. Ambient particulate matter exposure assessment

As air monitoring station data was not available in rural areas and there are no air monitoring stations on Nan’ao Island, we used well-established databases on ambient PM for PM assessment. The annual concentrations of PM_1_, PM_2.5_, and PM_10_ at a resolution of 1 km were estimated from ChinaHighPM_1_ dataset, ChinaHighPM_2.5_ dataset and ChinaHighPM_10_ dataset with a space–time extremely randomized trees model (denoted the STET model) (29–32), which combined information from multiple data sources, including satellite data (Multi-Angle Implementation of Atmospheric Correction (MAIAC) and aerosol product, meteorological, land cover, surface topographic and population data. Comprehensive utilization of various factors related to ambient PM ensured the accuracy of ambient PM assessment. Although air monitoring stations data or MAIAC data may be missing, the model can generally provide particle concentration for different seasons across China, which was available to assess ambient PM in Guangdong Province and Hebei Province (The spatial coverage was average value of 79%, more than 98% and more than 93% for PM_1_, PM_2.5_, and PM_10_). The out-of-station cross-validation coefficients of determination (CV-R^2^) were 0.77, 0.88, and 0.82 for PM_1_, PM_2.5_, and PM_10_, and, respectively. We calculated the three-year (2018–2020) average concentration before the baseline survey (2021) for each participant as the long-term exposure concentration of PM_1_, PM_2.5_, and PM_10_, and assigned PM concentration estimates for each participant according to their geocoded residential address. ArcGIS Desktop (version 10.2, ESRI Inc., Redlands, CA, United States) was used to describe the distribution of particulate matter.

### 2.3. Pulmonary outcome assessment

Trained and certified technicians carried out pulmonary function tests before and after bronchodilator inhalation using the same MasterScreen Pneumo PC spirometers (Jaeger, Germany) according to a standard protocol (33). Daily calibration with a 3-L syringe was performed for spirometers.

Pulmonary function tests were conducted from 8:00 to 12:00 a.m. each investigation day. We performed spirometric maneuvers with every participant in a seated position, wearing a nose clip, and using a disposable mouthpiece. Participants were required to perform up to eight forced expiratory maneuvers until the forced expiratory volume in 1 s (FEV_1_) and forced vital capacity (FVC) were reproducible within 150 ml. Inspiratory capacity (IC), and vital capacity (VC) were also tested. Predicted values of FEV1, FVC, and FEV_1_/FVC ratio can be automatically generated by system only based on Asian standards. So, using previously published spirometry reference values for the population aged 7–80 years in China as the reference (34) we calculated the predicted values of forced expiratory volume parameters to further ensure the quality control of pulmonary function. If any measured values/predicted values ratio appeared too low, it would be excluded. Finally, forced expiratory volume parameters were included in models as continuous variables.

### 2.4. Covariates

Potential confounders were included based on the previous studies on ambient PM and the respiratory system (18): (1) demographic and socioeconomic characteristics, including: age (years, as a continuous variable), age groups(20–29 years/30–39 years/40–49 years/50- years), height (cm, as a continuous variable), weight (kg, as a continuous variable), body mass index (BMI) categories(underweight/normal weight/overweight/obesity), residential area (urban/rural), highest education attained (illiterate or elementary school / high school / college or above), and annual personal income (≤50,000 RMB/>50,000 RMB); and (2) health-related lifestyle factors, including: smoking (no/yes), alcohol consumption (no/yes), and self-reported respiratory diseases history.

### 2.5. Statistical analysis

We characterized the distributions of all the covariates according to the means and standard deviations (SDs) or the medians and interquartile ranges (IQR) for continuous variables, and counts and percentages for categorical variables. Differences in baseline characteristics between participants in the present study and those in the overall CHCN-BTH were tested using Student’s *t*-test, the Wilcoxon rank sum test, or the chi-square test. Spearman rank correlation coefficients were determined to assess the relationship between sizes of particulate matter.

Difference in pulmonary function parameters, including the means and 95% confidence intervals (CIs), every 5-μg/m^3^ concentration increase in PM_1_, PM_2.5_, and PM_10_ were analyzed in multilevel models. As pulmonary function levels were substantially different among men and women in the two provinces and PM concentrations in Guangdong Province and Hebei Province have no overlap, we chose to conduct 4 multilevel models for men and women in different provinces, respectively. Individuals were treated as level 1 units, and different survey points (including Chenghai, Jinping, Nan’ao, Meizhou and Jiaoling in Guangdong Province and Baoding and Laiyuan in Hebei Province) were treated as level 2 units. Before analyses, a null model was fitted to test the random effects of the intercept term and evaluate whether the data was suitable for multilevel models. To ensure the comparability of the models, the same variables [age group (categorical variable), height (continuous variable), weight (continuous variable), education, annual personal income (categorical variable), smoking (categorical variable), alcohol consumption (categorical variable), and self-reported respiratory diseases (categorical variable)] were included in the models.

To identify the characteristics that might be significant in the association between ambient PM and pulmonary function, we performed stratified analyses according to BMI category (Under or Normal weight/ Overweight or Obesity) and age group (20–29 years/30–39 years/40–49 years/50-years). In addition, By performing analyses of participants without self-reported respiratory disease history, we conducted sensitivity analyses to assess the robustness of the associations between the 3 PM types and pulmonary function parameters.

All of the statistical analyses were performed using SAS version 9.4 (SAS Institute, Inc., Cary, NC). A *p*-value <0.05 was considered statistically significant for a two-tailed test.

## 3. Results

### 3.1. Descriptive statistic

The descriptive statistics for the main characteristics of the study participants are presented in Table 1. There were 1,592 participants included in this study (420 men and 481 women in Guangdong Province and 260 men and 431 women in Hebei Province). The demographic characteristics (including age, age groups, height and weight) of men and women were similar in Guangdong Province and Hebei Province. In comparison with Hebei Province, the participants in Guangdong Province had higher income and education levels and mostly lived in urban areas. The smoking rate among men in Guangdong Province (55.2%) was lower than that among men in Hebei Province (62.7%), and the alcohol consumption rate among men was 48.8% in Guangdong Province, which was lower than that in Hebei Province (69.2%). The rates among women in Guangdong Province were similar. The rates of self-reported respiratory diseases were lower among both men (1.9%) and women (1.3%) in Guangdong Province. The characteristics of pulmonary function of the study participants are summarized in Table 2. All pulmonary function parameters were lower in Guangdong Province than in Hebei Province, and all pulmonary function parameters were higher among men than among women. The means ± SDs for FEV_1_ were 3.4 ± 0.5 (L) and 2.6 ± 0.4 (L) for men and women, respectively, in Guangdong Province and 3.7 ± 0.6 (L) and 2.7 ± 0.4 (L) for men and women, respectively, in Hebei Province. The means ± SDs for FVC were 4.3 ± 0.6 (L) and 3.1 ± 0.5 (L) for men and women, respectively, in Guangdong Province and 4.7 ± 0.7 (L) and 3.4 ± 0.5 (L) for men and women, respectively, in Hebei Province.

**Table 1:** Characteristics of the study population in different provinces in DLCC (*n* = 1,592).

Characteristics	Guangdong			Hebei		
	Total (*n* = 901)	Men (*n* = 420)	Women (*n* = 481)	Total (*n* = 691)	Men (*n* = 260)	Women (*n* = 431)
*Age, mean ± SD, (years)*	41.5 ± 9.2	42.4 ± 10.1	40.7 ± 8.2	40.3 ± 9.1	40.2 ± 9.5	40.4 ± 8.9
*Age group, years, n (%)*						
20–29	106 (11.8)	50 (11.9)	56 (11.6)	97 (14.0)	37 (14.2)	60 (13.9)
30–39	248 (27.5)	110 (26.2)	138 (28.7)	222 (32.1)	85 (32.7)	137 (31.8)
40–49	420 (46.6)	163 (38.8)	257 (53.4)	267 (38.6)	96 (36.9)	171 (39.7)
50–73	127 (14.1)	97 (23.1)	30 (6.2)	105 (15.2)	42 (16.2)	63 (14.6)
*Height, mean ± SD, (cm)*	168.7 ± 5.8	168.7 ± 5.8	156.8 ± 5.4	163.1 ± 8.6	171.1 ± 5.9	158.5 ± 5.9
*Weight, mean ± SD, (kg)*	62.6 ± 12.4	68.6 ± 12.9	56.6 ± 8.8	67.5 ± 13.0	76.8 ± 12.3	61.9 ± 9.9
*BMI categories, n (%)^a^*						
Underweight	61 (6.8)	21 (5.0)	40 (8.3)	16 (2.3)	4 (1.5)	12 (2.8)
Normal weight	478 (53.0)	205 (48.8)	273 (56.8)	265 (38.4)	72 (27.7)	193 (44.8)
Overweight/Obesity	362 (40.2)	194 (46.2)	168 (34.9)	410 (59.3)	184 (70.8)	226 (52.4)
*Residential area, n (%)*						
Urban	550 (61.0)	286 (68.1)	264 (54.9	361 (52.2)	130 (50.0)	231 (53.6)
Rural	351 (39.0)	134 (31.9)	217 (45.1)	330 (47.8)	130 (50.0)	200 (46.4)
*Education, n (%)*						
Illiterate/Elementary school	61 (6.8)	27 (6.4)	34 (7.1)	70 (10.1)	23 (8.8)	47 (10.9)
High school	423 (47.0)	192 (45.7)	231 (48.1)	344 (49.8)	127 (48.8)	217 (50.3)
College or above	416 (46.2)	201 (47.9)	215 (44.8)	277 (40.1)	110 (42.3)	167 (38.7)
*Annual personal income (CHY), n (%)*						
≤50000RMB/year	441 (49.2)	147 (35.2)	294 (61.4)	495 (71.8)	146 (56.6)	349 (81.0)
>500000RMB/year	456 (50.8)	271 (64.8)	185 (38.6)	194 (28.2)	112 (43.4)	82 (19.0)
*Smoking, n (%)*						
No	657 (72.9)	188 (44.8)	469 (97.5)	521 (75.4)	97 (37.3)	424 (98.4)
Yes	244 (27.1)	232 (55.2)	12 (2.5)	170 (24.6)	163 (62.7)	7 (1.6)
*Alcohol consumption, n (%)*						
No	663 (73.6)	215 (51.2)	448 (93.1)	486 (70.3)	80 (30.8)	406 (94.2)
Yes	238 (26.4)	205 (48.8)	33 (6.9)	205 (29.7)	180 (69.2)	25 (5.8)
*Self-reported respiratory diseases, n (%)*	14 (1.5)	8 (1.9)	6 (1.3)	27 (3.9)	8 (3.1)	19 (4.4)

Self-reported respiratory diseases include tracheitis, bronchitis, pneumonia, asthma, Chronic obstructive pulmonary disease, etc.

DLCC, Diverse Life-Course Cohort; N, number; SD, standard deviation. Underweight was defined as BMI < 18.5 kg/m^2^, 18.5 ≤ BMI < 24 as normal weight, 24 ≤ BMI < 28 as overweight, and BMI ≥ 28 as obesity. BMI body mass index, kg/m^2^.

**Table 2:** Pulmonary function characteristics of study population in different provinces in DLCC (*n* = 1,592).

Variable	Guangdong				Hebei				*p*-value for province
	Total (*n* = 901)	Men (*n* = 420)	Women (*n* = 481)	*p*-value for gender	Total (*n* = 691)	Men (*n* = 260)	Women (*n* = 431)	*p*-value for gender	
VC, mean ± SD, (L)	3.8 ± 0.8	4.4 ± 0.6	3.2 ± 0.5	<0.0001	4.0 ± 0.9	4.8 ± 0.7	3.4 ± 0.5	<0.0001	<0.0001
IC, mean ± SD, (L)	2.4 ± 0.7	2.7 ± 0.70	2.1 ± 0.5	<0.0001	2.6 ± 0.7	3.1 ± 0.7	2.2 ± 0.5	<0.0001	<0.0001
FEV_1_, mean ± SD, (L)	3.0 ± 0.6	3.4 ± 0.5	2.6 ± 0.4	<0.0001	3.1 ± 0.7	3.7 ± 0.6	2.7 ± 0.4	<0.0001	0.0054
FVC, mean ± SD, (L)	3.7 ± 0.8	4.3 ± 0.6	3.1 ± 0.5	<0.0001	3.9 ± 0.9	4.7 ± 0.7	3.4 ± 0.5	<0.0001	<0.0001
FEV_1_/FVC, mean ± SD, (%)	81.4 ± 7.1	80.0 ± 7.2	82.6 ± 6.9	<0.0001	79.5 ± 7.3	77.5 ± 7.3	80.7 ± 7.0	<0.0001	<0.0001
FEV_1_%predicted, mean ± SD, (%)	101.4 ± 11.8	100.5 ± 12.1	102.3 ± 11.5	0.0183	104.6 ± 12.7	103.0 ± 12.3	105.6 ± 12.8	0.0086	<0.0001
FVC %predicted, mean ± SD, (%)	101.0 ± 12.3	100.1 ± 12.3	101.8 ± 12.2	0.0442	106.7 ± 13.1	105.9 ± 13.1	107.2 ± 13.0	0.1885	<0.0001
FEV_1_/FVC%predicted, mean ± SD, (%)	100.4 ± 8.0	100.2 ± 8.4	100.7 ± 7.7	0.3608	98.1 ± 8.3	97.2 ± 8.5	98.6 ± 8.1	0.0350	<0.0001

VC, vital capacity; IC, inspiratory capacity; FEV_1_, forced expiratory volume in 1 s; FVC, forced vital capacity; N, number; SD, standard deviation.

**Figure 1:**
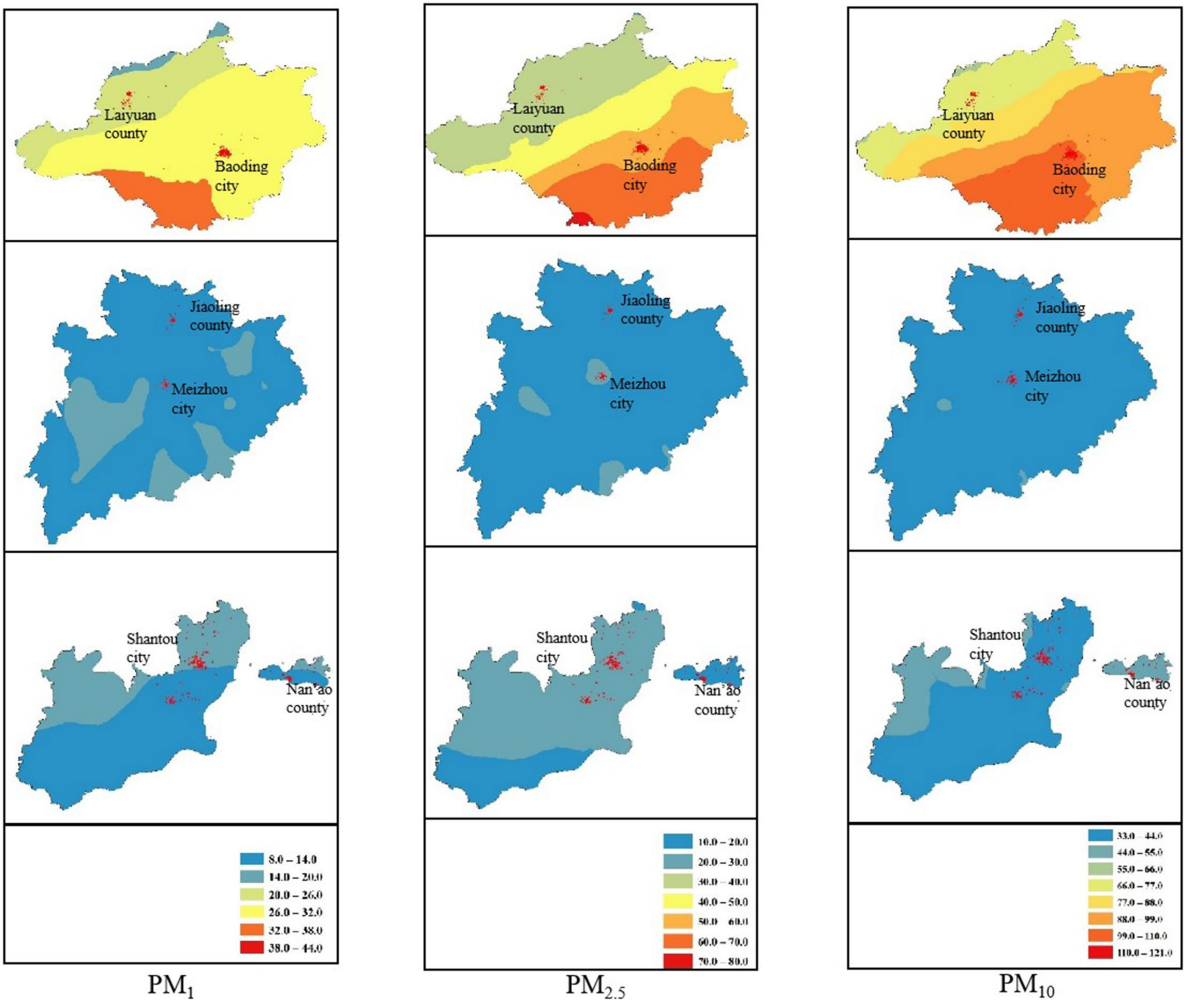
Locations of participant’s residence and 3-year average of PM_1_, PM_2.5_, and PM_10_ concentrations from 2018 to 2020 in Shantou and Meizhou, Gaungdon Province and Baoding Hebei, Province.

The distributions of the 3-year average particulate matter concentrations and Spearman rank correlation coefficients from 2018 to 2020 are summarized. The PM_1_, PM_2.5_, and PM_10_ concentrations varied greatly across study sites with medians (IQRs) of 15.4 (1.2) μg/m^3^, 24.4 (0.9) μg/m^3^, and 41.2 (1.5) μg/m^3^ in Shantou and Meizhou, Guangdong Province and 33.0 (9.1) μg/m^3^, 57.0 (22.0) μg/m^3^, and 98.9 (28.3) μg/m^3^ in Baoding, Hebei Province, respectively. The 3-year average concentrations of PM_1_, PM_2.5_, and PM_10_ in Baoding were much greater than two times those in Shantou and Meizhou. With higher concentrations, the three ambient PM all showed a gradual upward trend from northwest to southeast in Baoding; however, the ambient PM concentrations were low and evenly distributed in Shantou and Meizhou, as shown in Figure 1. In addition, the 3-year average concentrations of the 3 PM types in Baoding city were much higher than those in Laiyuan county. Compared with Shantou and Meizhou, there were stronger correlations between the three kinds of PM in Baoding (Spearman rank correlation coefficients were 0.8, 0.8, and 0.9 in Baoding and 0.4, 0.5, and 0.9 in Shantou and Meizhou).

### 3.2. Associations between ambient PM and pulmonary function parameters

The associations of the 3 PM types with pulmonary function parameters are summarized. The discrepancy in the relationship between the 3 PM types and pulmonary function in Guangdong Province and Hebei Province was reflected in forced expiratory volume parameters. In Hebei Province, higher concentrations of PM showed significant associations with lower levels of FVC and higher levels of FEV_1_/FVC among men, with a 5-μg /m^3^ increase in PM_1_, PM_2.5_, and PM_10_, decreases in FVC of 122.1 mL (95% CI: 30.8, 213.4), 54.6 ml (95% CI: 15.8, 93.3) and 42.9 mL (95% CI: 12.7, 73.1) and increases in FEV_1_/FVC of 2.2% (95% CI: 0.6, 3.9), 1.1% (95% CI: 0.4, 1.9), and 0.9% (95% CI: 0.3, 1.5). Among women, higher concentrations of PM showed significant associations with lower levels of FEV_1_ and FVC, with a 5-μg /m^3^ increase in PM_1_, PM_2.5_, and PM_10_, decreases in FEV_1_ of 51.1 ml (95% CI: 9.7, 92.4), 21.6 ml (95% CI: 4.3, 38.9) and 16.7 ml (95% CI: 3.3, 30.1), respectively, and decreases in FVC of 77.8 ml (95% CI: 10.0, 145.6), 38.7 ml (95% CI: 9.0, 68.5) and 31.1 ml (95% CI: 8.1, 54.1), respectively. The effect on forced expiratory volume parameters was magnified with the decrease in PM size, and the association between ambient PM and FVC were greater than that with FEV_1_ in Hebei Province. There were no statistically significant associations between the three ambient PM types and forced expiratory volume parameters in Guangdong Province.

### 3.3. Stratified analyses and sensitivity analyses

**Figure 2:**
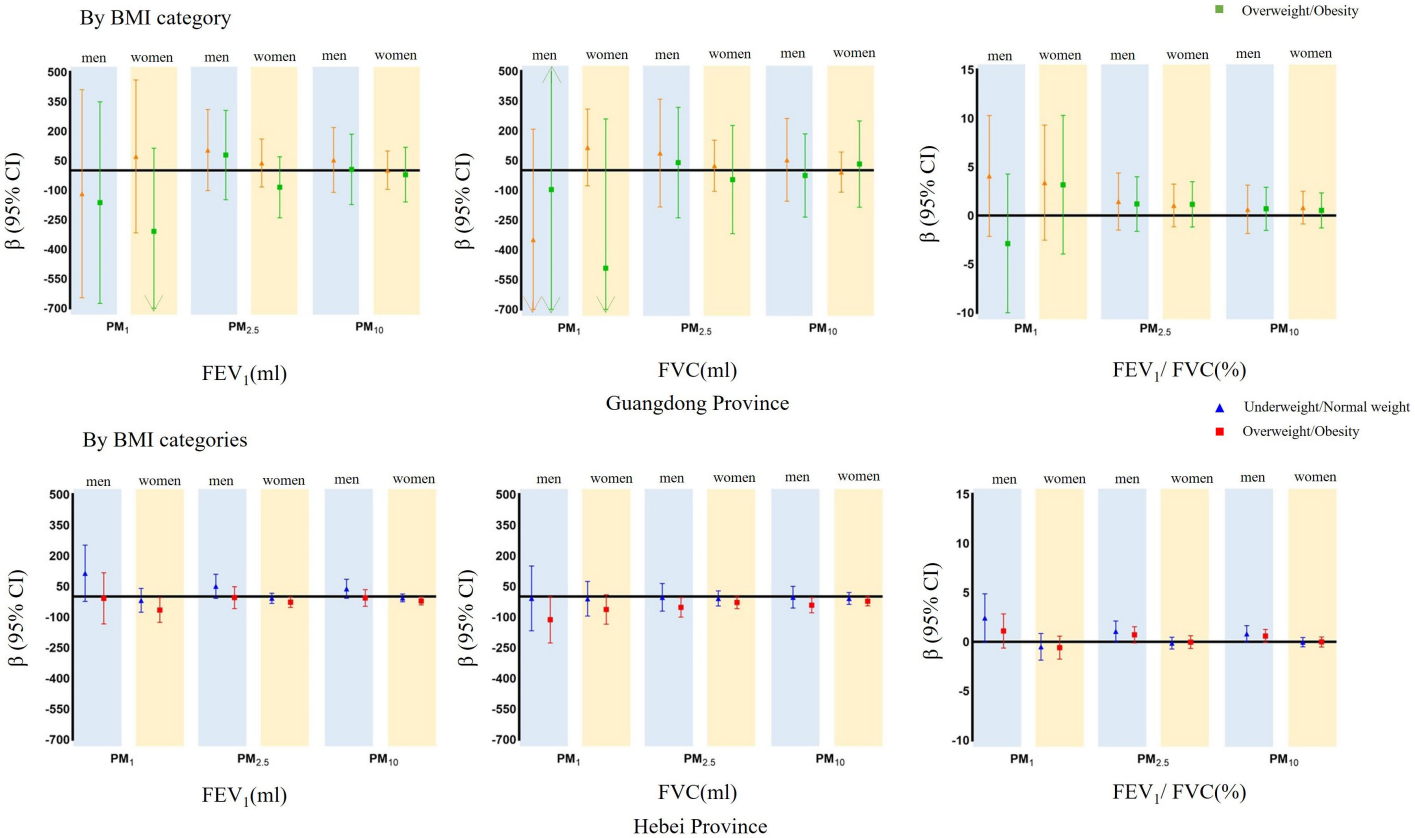
Mean differences with 95% confidence intervals in the pulmonary function parameters every 5 μg/m^3^ increase of PM_1_, PM_2.5_, and PM_10_ concentrations, stratified analyzed BMI categories in Guangdong and Hebei Provinces. The blue shadow represents men and the yellow shadow represents women.

**Figure 3:**
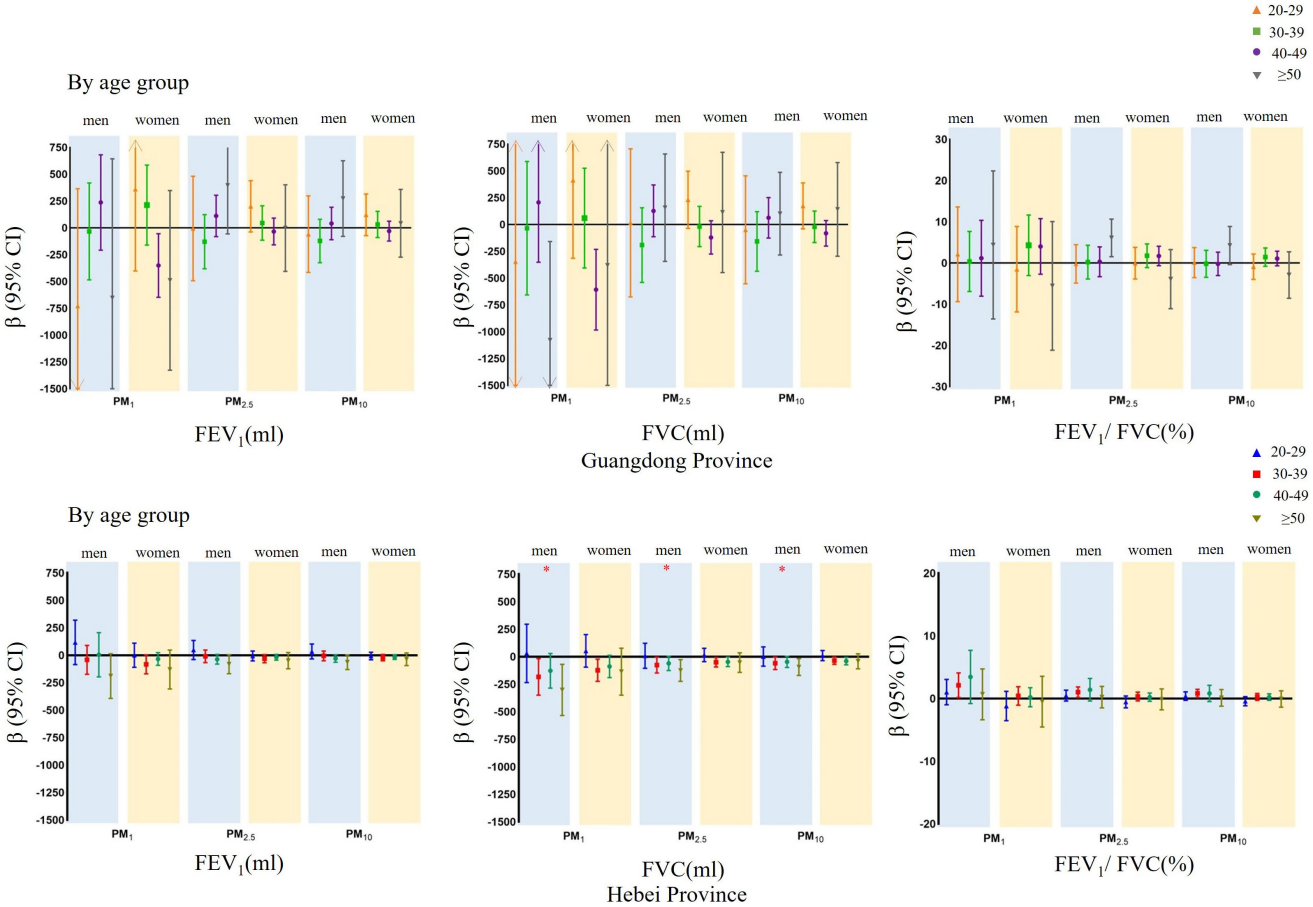
Mean differences with 95% confidence intervals in the pulmonary function parameters every 5 μg/m^3^ increase of PM_1_, PM_2.5_, and PM_10_ concentrations, stratified analyzed by age group in Guangdong and Hebei Provinces. The blue shadow represents men and the yellow shadow represents women. *P for interaction terms with statistically significance between air pollutants and modifiers.

The associations between ambient PM and forced expiratory volume parameters stratified by BMI category and age group are presented in Figures 2, 3. In the BMI category-stratified analysis, the adverse associations between forced expiratory volume parameters and ambient PM seemed predominant in overweight and obese populations, while the difference were not significant. In the age group-stratified analysis, the impact of PM on pulmonary function increased with age and greater associations between ambient PM and FVC in the age groups of 30–40 years old and those over 50 years old were observed both in men and women, however there was only an interaction between age and ambient PM on FVC among men that was not present among women in Hebei Province. Similarly, adverse association of PM on FEV_1_ increased with age and the effect was more pronounced in the age groups of over 50 years old. The positive association between PM and FEV_1_/FVC ratio increased with age in the age group of 20–50 years, and declined in group aged over 50 years. While, there was no interaction between age and ambient PM on FEV_1_ and FEV_1_/FVC ratio.

**Figure 4:**
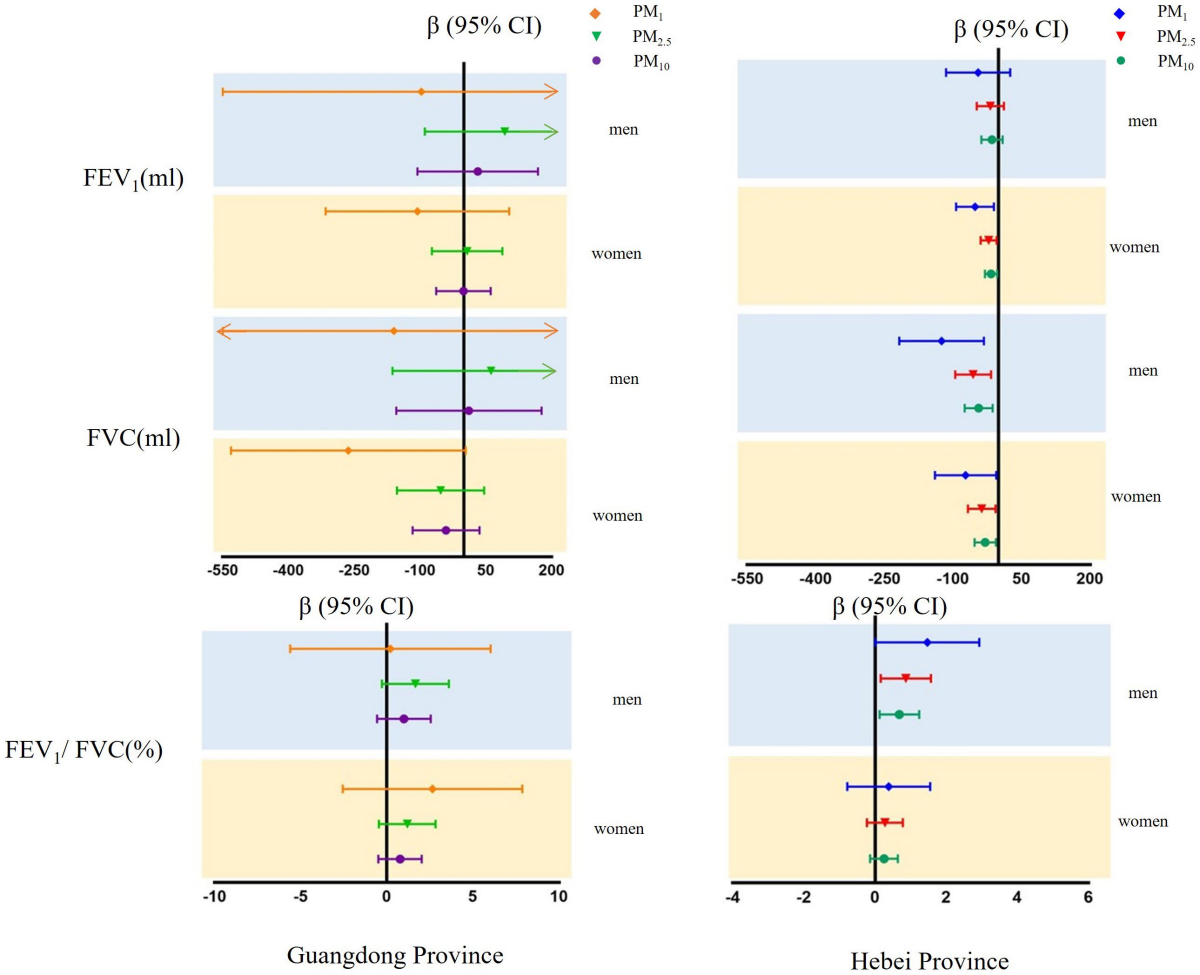
Mean differences with 95% confidence intervals in the pulmonary function parameters every 5 μg/m^3^ increase of PM_1_, PM_2.5_, and PM_10_ concentrations, sensitivity analyzed in participants without self-respiratory disease in Guangdong and Hebei Provinces. The blue shadow represents men and the yellow shadow represents.

The results of sensitivity analyses are presented in Figure 4. No changes occurred to the associations of PM_1_, PM_2.5_, or PM_10_ with forced expiratory volume parameters when we analyzed data among participants without self-reported respiratory diseases.

## 4. Discussion

The DLCC was a large-scale prospective study to explore the long-term effects of ambient air pollutants or other risk factors on population health of all ages. In the baseline survey of the CHB cohort (Visit 2 of the DLCC), we discovered that the 3-year average concentrations of ambient PM in Baoding city were much higher than those in Laiyuan county and two times higher than those in Meizhou and Shantou. Previous studies have illustrated that the spatial distributions of ambient PM vary greatly at different pollution levels across China (35) and we also observed this phenomenon. In Shantou and Meizhou, not only was the level of ambient PM low, but the correlations between ambient PM types was weaker. Due to high vegetation coverage and/or proximity to the ocean, the structure of ambient PM types was simple. In contrast, in Baoding, there were high concentrations of PM_1_, PM_2.5_, and PM_10_, which were strongly correlated. A gradient increase in ambient PM was observed from the mountains (northwest Baoding) to the urban areas (southeast Baoding). Similar trends showed that the potential sources of ambient PM may be the same. Coal consumption and well-developed traffic have brought ambient particulate matter pollution to Baoding City.

Based on our study, we discovered negative effects of long-term ambient PM exposure on FVC and positive effects on FEV_1_/FVC among men and adverse associations between ambient PM and FEV_1_, FVC among women at high levels of PM. Stable effects were shown in sensitivity analyses. Moreover, higher estimated effects were observed for PM_1_, than for PM_2.5_ and PM_10_. Particle size determines how deep particles can penetrate into the lung compartments. PM_2.5-10_(particulate matter with diameters between 2.5 and 10 μm)is mainly deposited in the upper airways and can be cleared by the mucociliary system. PM_2.5_ deposits in the tracheobronchial region, whereas PM_1_ can reach the lung periphery, i.e., the alveolar region (U.S. Environmental Protection Agency) (36). The deeper PM is deposited, the slower it is removed, and the more likely it is to cause cell damage (37). PM_2.5_ has the ability to penetrate deeper into the respiratory tract than PM_10_, where PM_2.5_ can more easily penetrate the air-blood barrier; while, greater health risks may be associated with PM_1_ exposure, because it can access the gas-exchange region of the lungs (38, 39). In addition, smaller PM has a higher surface area to volume ratio, which having greater potential for deleterious biological interactions with respiratory tissues and risks for adverse health outcomes (40). Moreover, the effects of PM on pulmonary function may be related to induced airway inflammation, alveolar inflammation and lung tissue damage (40, 41). Therefore, finer PM is more strongly associated with reduced pulmonary function.

In our study, associations of ambient PM with pulmonary function differed among men and women in different regions, and it was suggested that when investigating risk factors for pulmonary function, regions and sex should be classified. Our study findings about the association of high concentration of PM_2.5_ and PM_10_ with FEV_1_ and FVC were similar to those of previous large-scale epidemiological studies (18–20, 42, 43). In contrast, most studies showed that PM_2.5_ and PM_10_ was related to decreased FEV_1_/ FVC, while we failed to find the negative associations among women and found positive associations between ambient PM and FEV_1_/ FVC among men. This may be ascribable to the differences in the concentration of PM_2.5_ and PM_10_, climatic conditions, geographical conditions and population susceptibility. Studies that found a larger effect in the extent of ambient PM on FVC than FEV_1_ did not find a significant association with FEV_1_/FVC (20). Although the effect of PM associated with pulmonary function decline differed between men and women, restrictive effects of ambient PM were observed. Restrictive pattern pulmonary function is generally characterized by a reduced FVC and/or FEV_1_, but a stable or higher FEV_1_/FVC (44). In a cross-sectional study in Shanghai, restrictive ventilation patterns of PM_2.5_ were also observed (45). We discovered that the association between PM_2.5_ and FVC was greater among men than among women in our study, and both were greater than that in Shanghai. The PM_2.5_ concentration in western Shanghai between 2013 and 2014 was similar to that in Baoding city from 2018 to 2020, while, compared with 2013, ambient PM decreased by more than 40% in 2018 in Hebei Province (21). Thus, long-term exposure to ambient PM at higher concentrations is probably associated with more serious restrictive lung impairment and ambient PM-related restrictive ventilatory dysfunction suggests the presence of pulmonary fibrosis in Hebei Province. In addition, restrictive ventilation patterns were not only associated with respiratory diseases but also with cardiovascular disease (CVD) and mortality (46, 47). We should pay attention to restrictive pulmonary patterns in areas similar to Baoding city. For PM_1_, a similar restrictive effect was shown in general population, while the magnitude of the association was larger than that for PM_2.5_ and PM_10_.

In BMI category-stratified analysis, the overweight population was affected more by ambient PM in the ESCAPE study and the CPH study (18, 43, 45). However, we failed to find a significantly different association between ambient PM and large airway function in different BMI categories and we only observed a different trend, possibly because breathlessness and grunting may occur in overweight and obese people and they may take steps to control weight. In age group-stratified analysis, we found that the impact of PM on FVC increased with age and FVC was susceptible to ambient PM among men aged 30–39 years and over 50 years. As was reported, age over 40 years is a risk factor for COPD (48) and the pulmonary function of adults reaches a peak at approximately 25 years and then began to decline (33). The aging-related decline in pulmonary function may be expedited by ambient PM. A previous report showed that the smoking rate among men in Hebei Province was high (49). In our study, we also found that unhealthy lifestyle habits such as smoking and drinking are common among men in Hebei Province. Such unhealthy lifestyle habits together with long-term exposure to ambient PM were likely to accelerate restrictive ventilatory dysfunction with increasing age among men in Hebei Province. Older men in Hebei Province should focus on respiratory health. However, the interaction between BMI categories, age groups and ambient PM on large airway function needs to be further studied by expanding the sample size. Presently, ambient PM pollution in Baoding city is still very serious. The Baoding government needs to take stricter measures to control air pollution. Ambient PM seems to have a greater restrictive effect on men, especially older men. Stricter smoke control measures need to be taken to prevent ambient PM from aggravating restrictive ventilatory dysfunction as men age. In addition to screening for ventilatory dysfunction, it is necessary to include pulmonary function examinations in the routine physical examination of people aged 40 and above. In this way, the incidence of chronic obstructive pulmonary disease, restrictive pulmonary disease and CVD can be reduced among older adults. For patients with ventilatory dysfunction, regular follow-ups with close attention to cardiopulmonary status should be carried out in areas with high concentrations of ambient PM.

## 5. Strengths and limitations

Our study has several strengths. Different natural ambient PM patterns were selected to conduct this comparative studies in South China and North China. We are also the first to conduct research on the relationship between ambient PM and lung function on Nan’ao Island. Shantou and Meizhou are typical areas with low ambient PM concentrations for decades, and ambient PM concentration in Baoding city has remained consistently high for several decades. Although we only evaluated ambient PM concentrations for 3 years, it can represent a long-term stable exposure. Our results can better reflect the association between different long-term ambient PM patterns and lung function. Besides, the ambient PM concentration in Laiyuan county was far lower than that in Baoding city, and clean controls were obtained to conduct comparative studies in the same areas. We could better observe the relationship between ambient PM and pulmonary function in Baoding. Nonetheless, this study also has the following limitations. First, there were few air monitoring stations in rural areas and we assigned PM concentration estimates for each participant based only on their geocoded current addresses; thus, exposure misclassification errors were inevitable. Nevertheless, we estimated the residential concentrations by using models with good performance in external cross validation. Second, because indoor ambient PM data were not available, we only included ambient PM data. Third, because it was time consuming to obtain pulmonary function measurement, we were unable to obtain more samples during this study. Finally, a questionnaire was used to collect self-reported demographic information and lifestyle characteristics, and thus, recall bias might have occurred.

## 6. Conclusion

In conclusion, long-term exposure to different levels of ambient PM was associated with large airway function differently. High concentrations of PM_1_, PM_2.5_, and PM_10_ were closely associated with decreased FVC and increased FEV_1_/ FVC among men and reduced FEV_1_ and FVC among women, greater effects were observed for PM_1_, followed by PM_2.5_ and PM_10_. The restrictive effects of ambient PM on men and older adults should be of greater concern.

### Collaboration

The collaborators are: Guangdong Provincial People’s Hospital, Hebei University, China-Japan Friendship Hospital, Institute of Respiratory Medicine, Chinese Academy of Medical Sciences, National Clinical Research Center for Respiratory Diseases.

## Data availability statement

The data analyzed in this study is subject to the following licenses/restrictions: the data underlying this article will be shared on reasonable request to the corresponding author. Requests to access these datasets should be directed to guangliang_shan@163.com.

## Ethics statement

The studies involving human participants were reviewed and approved by the Bioethical Committee of Institute of Basic Medical Sciences, Chinese Academy of Medical Sciences (055–2020). The patients/participants provided their written informed consent to participate in this study.

## Author contributions

GS and QL contributed to the study conception and design and performed the data analysis. All authors contributed to the material preparation, data collection, and data interpretation. The first draft of the manuscript was written by QL and all authors commented on previous versions of the manuscript. All authors read and have approved the submitted version. All authors have agreed both to be personally accountable for the author’s own contributions and to ensure that questions related to the accuracy or integrity of any part of the work.

## Funding

This study is support by the National Key R&D Program of China (2016YFC0900600) and CAMS Innovation Fund for Medical Sciences (CIFMS; 2020-I2M-2–009/2021-I2M-1-023).

## Conflict of interest

The authors declare that the research was conducted in the absence of any commercial or financial relationships that could be construed as a potential conflict of interest.

## Publisher’s note

All claims expressed in this article are solely those of the authors and do not necessarily represent those of their affiliated organizations, or those of the publisher, the editors and the reviewers. Any product that may be evaluated in this article, or claim that may be made by its manufacturer, is not guaranteed or endorsed by the publisher.
